# Supplementary figures and images for: Bet-hedging antimicrobial strategies in macrophage phagosome acidification drive the dynamics of Cryptococcus neoformans intracellular escape mechanisms
Source: PLoS Pathog. 2022 Jul 11;18(7):e1010697. doi: 10.1371/journal.ppat.1010697 (PMC9302974; doi:10.1371/journal.ppat.1010697)

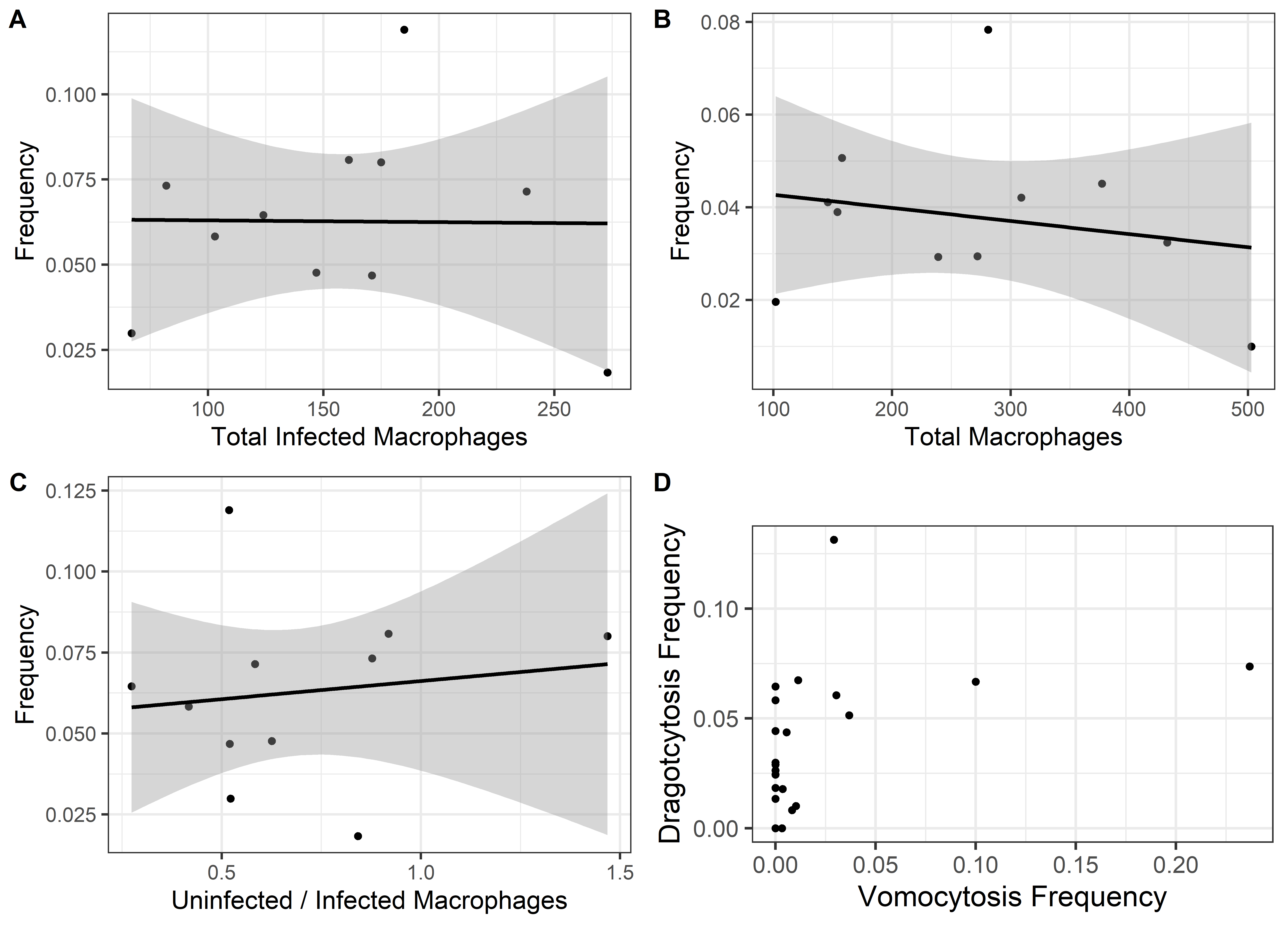

Supplement: S1 Fig — A. Dragotcytosis frequency did not correlate with total number of infected macrophages. B. Dragotcytosis frequency did not correlate with the density of total macrophages. C. Dragotcytosis frequency did not correlate with the proportion of uninfected to infected macrophages. Linear regressions were performed on each dataset with 95% confidence intervals (gray). D. Frequencies of Vomocytosis and Dragotcytosis obtained from 22 independent movies of C. neoformans strain H99 infection of macrophages. The Pearson correlation between the two processes is 0.41 with P = 0.055 and the Spearman correlation is 0.48 with P = 0.025. (TIFF) [file ppat.1010697.s001.tiff]

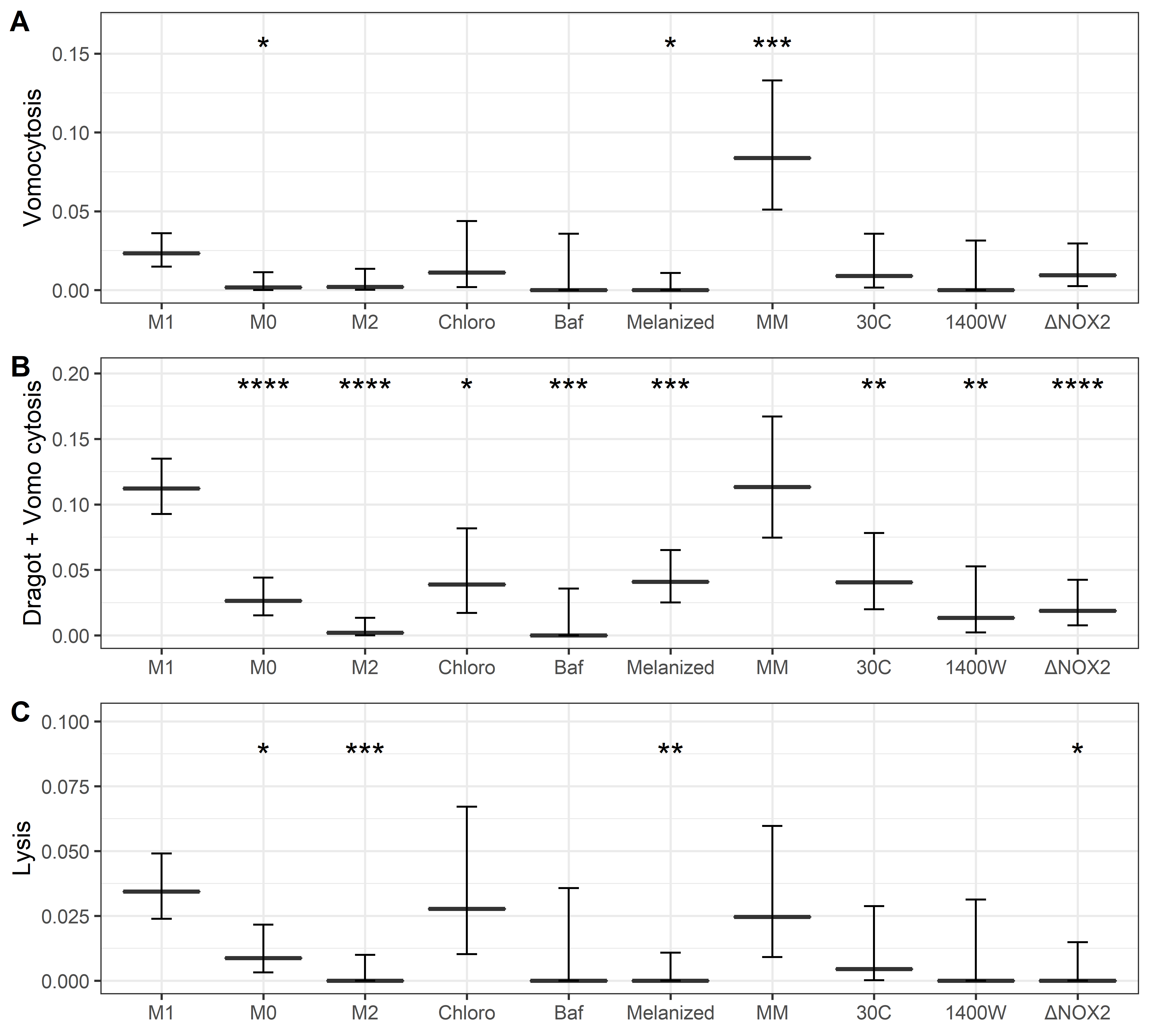

Supplement: S2 Fig — A. Vomocytosis frequency among infected macrophages. B. Combined frequency of Dragotcytosis and Vomocytosis among infected macrophages. C. Lysis frequency among infected macrophages. All conditions have similar frequency. Event frequencies of wild-type KN99α strain and mutant C. neoformans after ingestion by M1 polarized BMDMs. *, **, ***, **** signify P < 0.05, 0.01, 0.001, and 0.0001 via test of equal proportions, respectively. Bonferroni correction was applied for multiple hypotheses. Boxplots signify median with 95% confidence interval tails. Data was gathered from n of 901, 569, 478, 180, 130, 440, 203, 222, 149, and 619 for M1, M0, M2, Chloroquine (Chloro), Bafilomycin (Baf), Melanized, Capsule Induced (MM), 30C, 1400W, and ΔNOX2 respectively. (TIFF) [file ppat.1010697.s002.tiff]

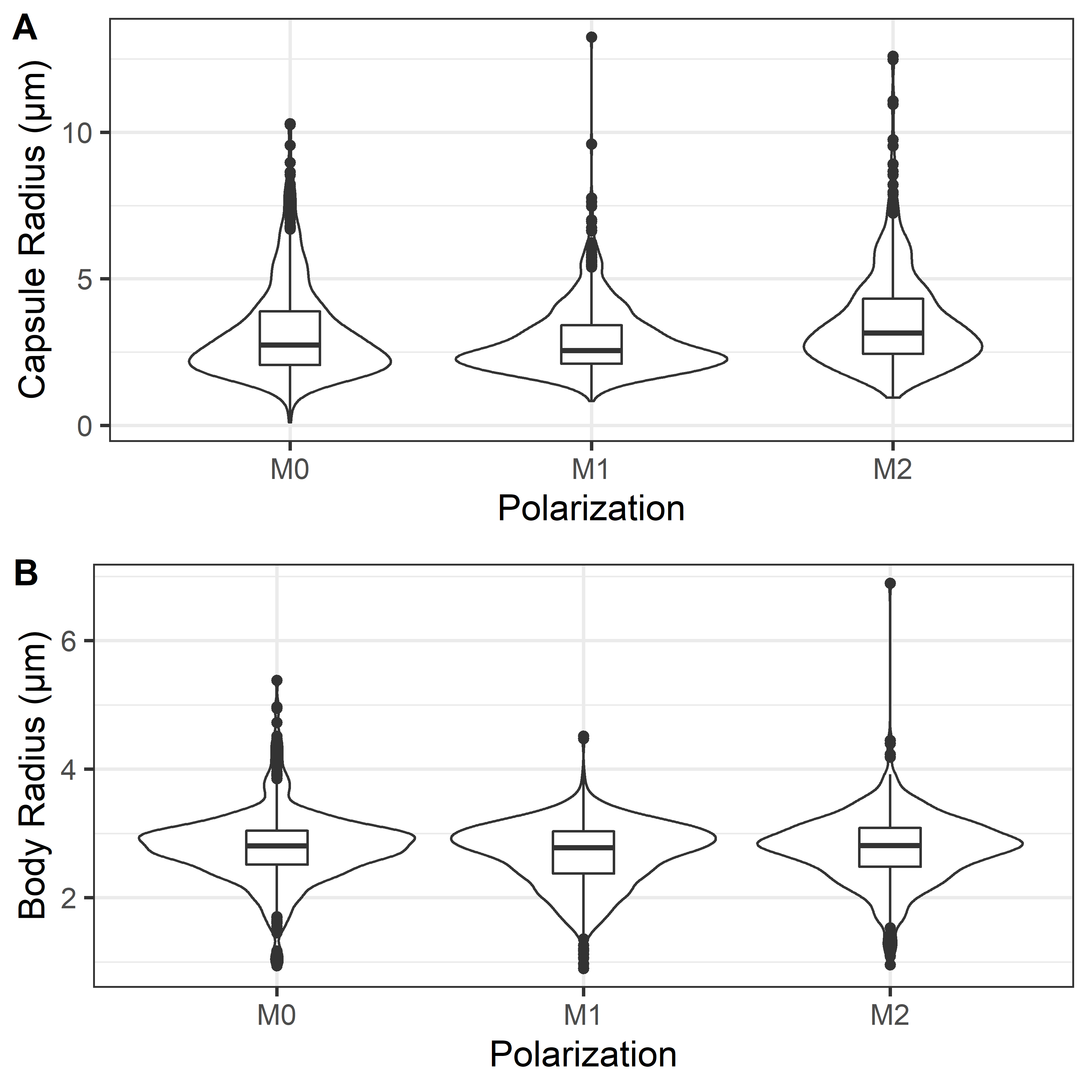

Supplement: S3 Fig — Capsules and cell bodies are measured by preparing and imaging India Ink slides and a previously published[20] measuring code. No significant differences were found between the polarization states of host macrophages. Boxplots signify median with 95% confidence interval tails. For each experiment, data was collected for at least three independent experiments and total n of 563, 870, and 554 for M0, M1, and M2 respectively. (TIFF) [file ppat.1010697.s003.tiff]

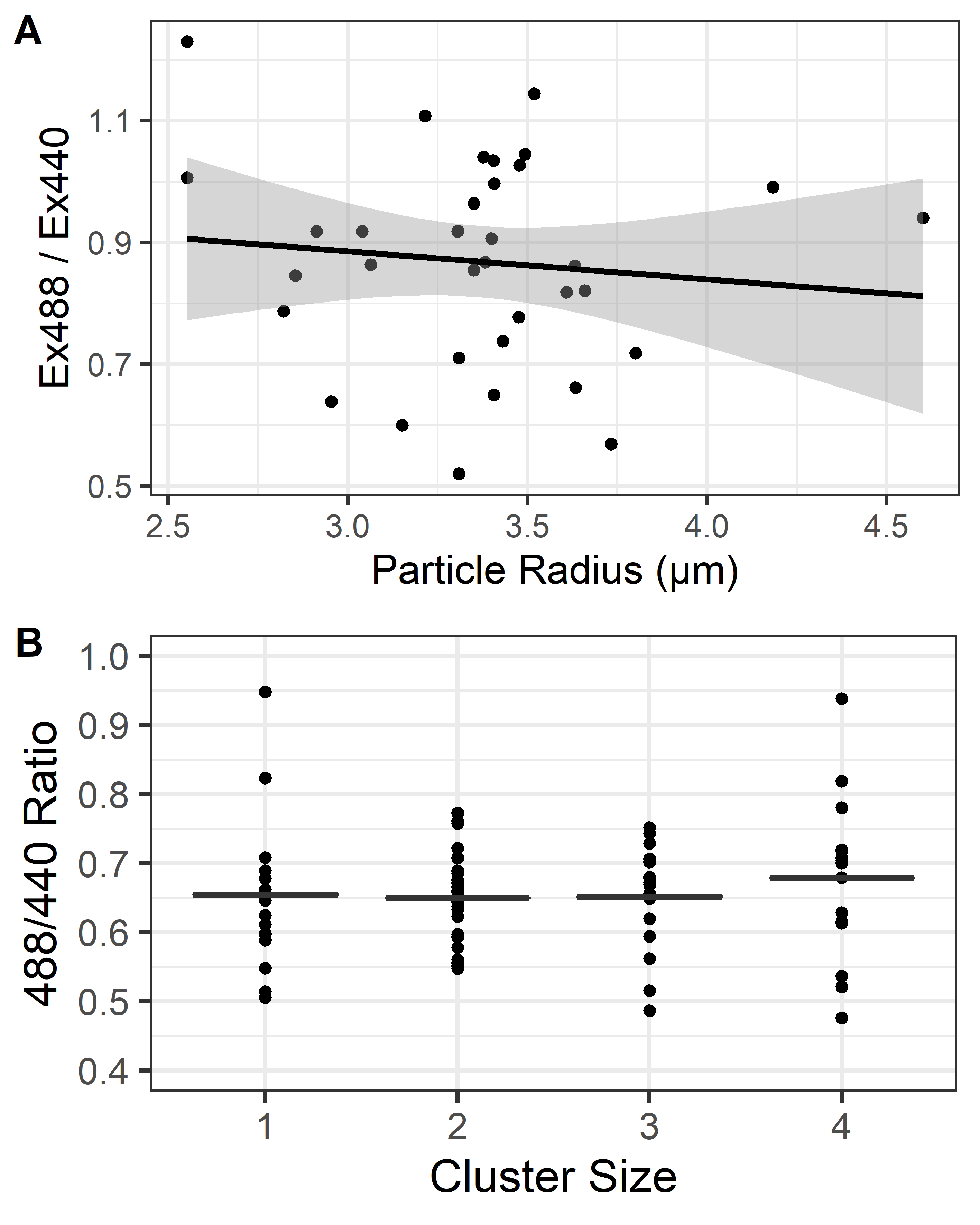

Supplement: S4 Fig — A. Fluorescence ratio of ingested C. neoformans. We found no significant correlation. B. Fluorescence ratio of ingested inert latex beads of 0.6 μm diameter. Even with a cluster of four particles within a single phagosome we did not detect a threshold at which size alone disrupts the phagolysosomal pH. Boxplots signify median with 95% confidence interval tails and n of 16, 30, 18, 16 for cluster sizes 1, 2, 3, and 4 respectively. (TIFF) [file ppat.1010697.s004.tiff]

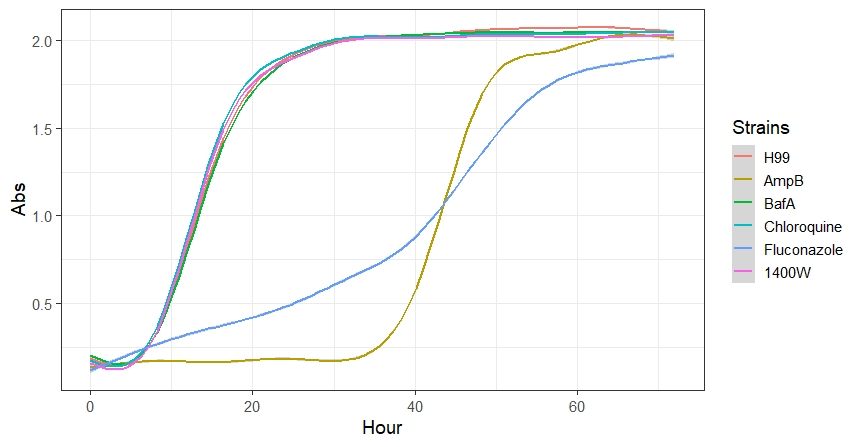

Supplement: S5 Fig — Cultures were seeded at 104 cells / mL and grown for 72 h at 30°C with 120 rpm shaking. Fluconazole and Amphotericin B inhibit cryptococcal growth while Bafilomycin, Chloroquine, and 1400W do not. Lines with shaded areas represent 95% CI of 3 independent replicates. (JPEG) [file ppat.1010697.s005.jpeg]

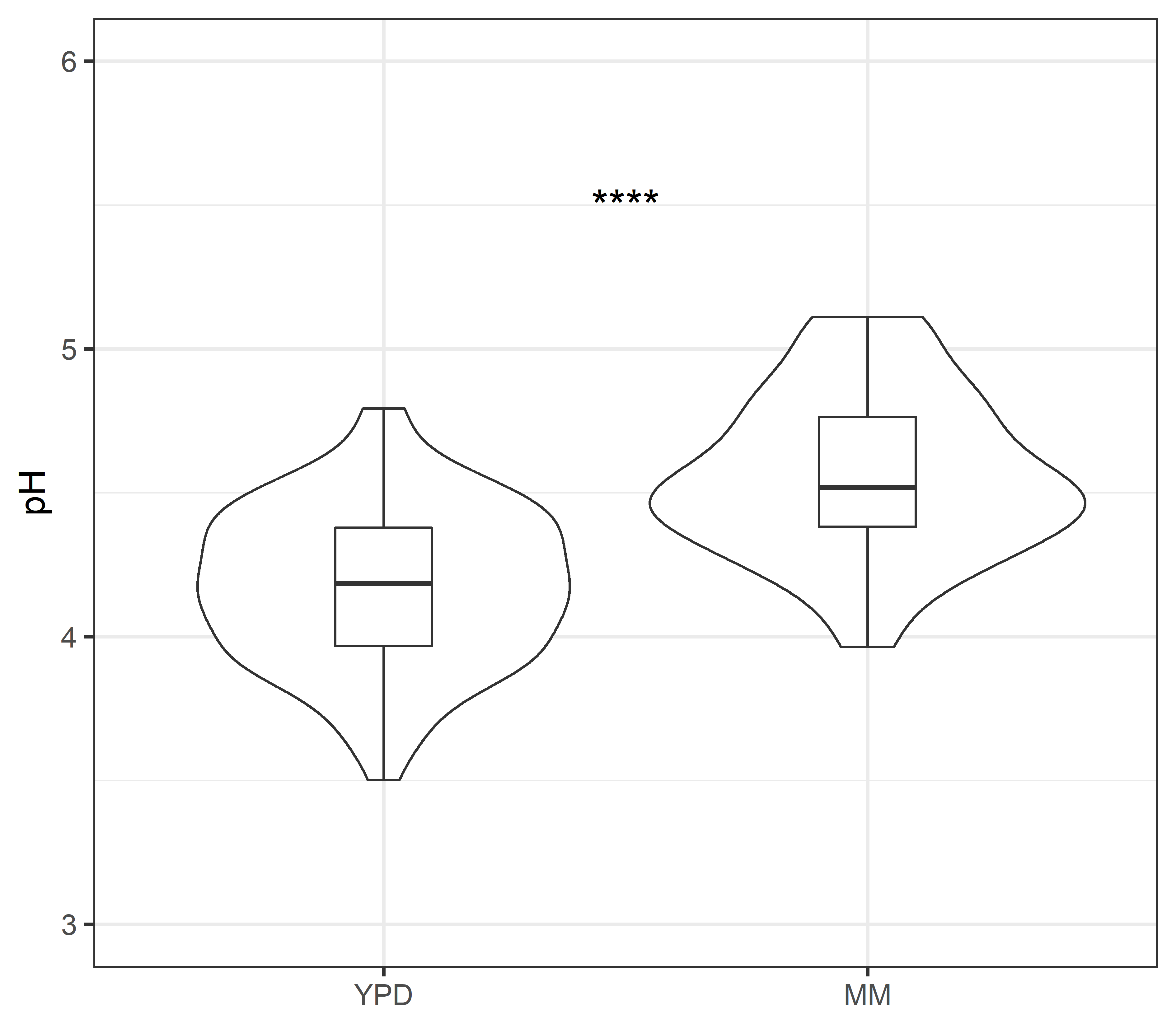

Supplement: S6 Fig — Data was collected from three independent experiments and total n = 83 and 63 phagolysosomes, respectively. **** signifies P < 0.0001 via two tailed t-test. Boxplots signify median with 95% confidence interval tails. (TIFF) [file ppat.1010697.s006.tiff]

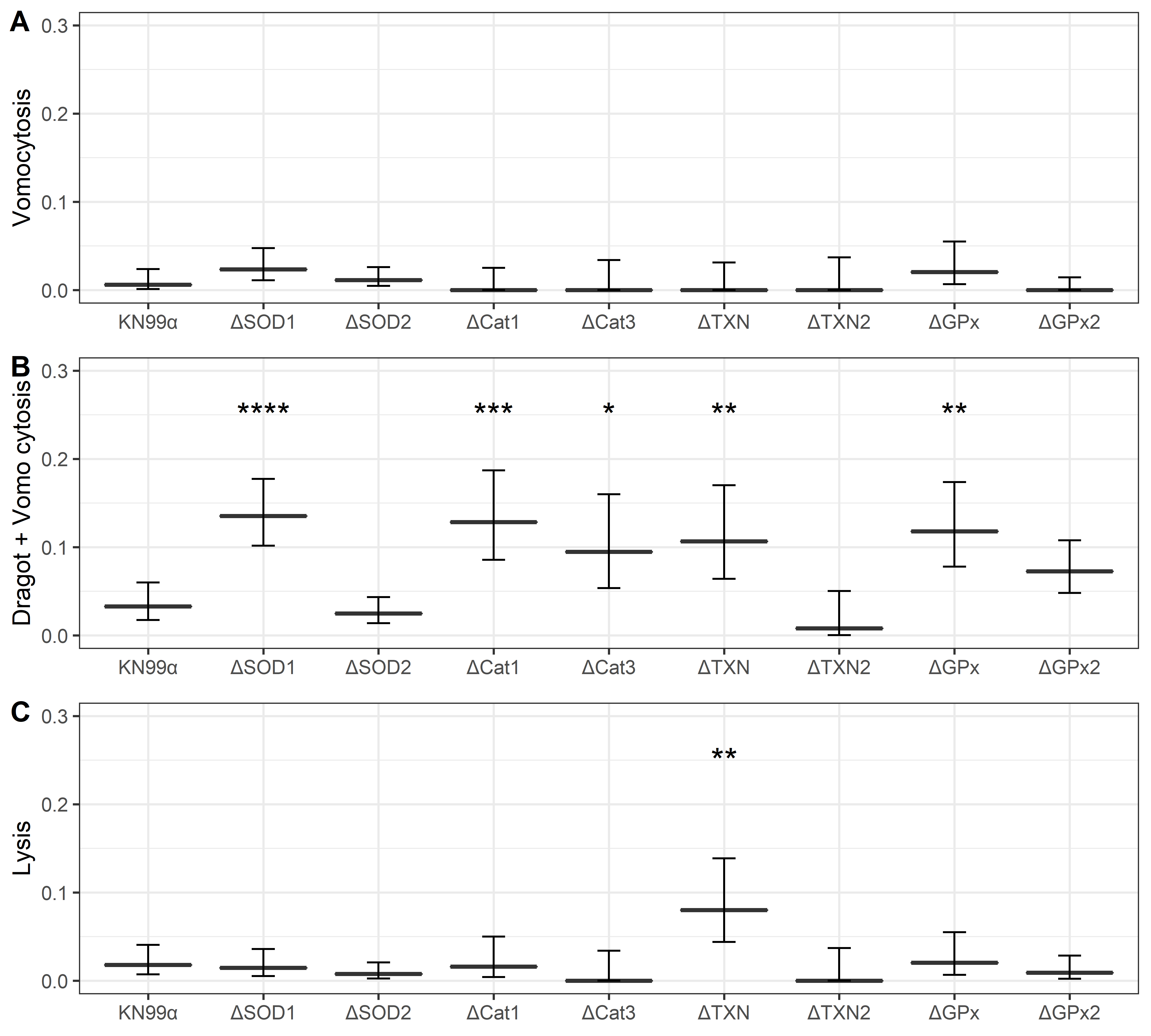

Supplement: S7 Fig — A. Vomocytosis frequency among infected macrophages B. Combined Dragotcytosis and Vomocytosis frequency among infected macrophages. C. Lysis frequency among infected macrophages. Graphs depict means with 95% confidence intervals. *, **, ***, **** signify P < 0.05, 0.01, 0.001, and 0.0001 via test of equal proportions, respectively. Bonferroni correction was applied for multiple hypotheses. Boxplots signify median with 95% confidence interval tails. Data was gathered from n of 333, 340, 522, 187, 137, 150, 125, 195, and 330 for KN99α, ΔSOD1, ΔSOD2, ΔCat1, ΔCat3, ΔTXN, ΔTXN2, ΔGPx, and ΔGPx2 respectively. (TIFF) [file ppat.1010697.s007.tiff]

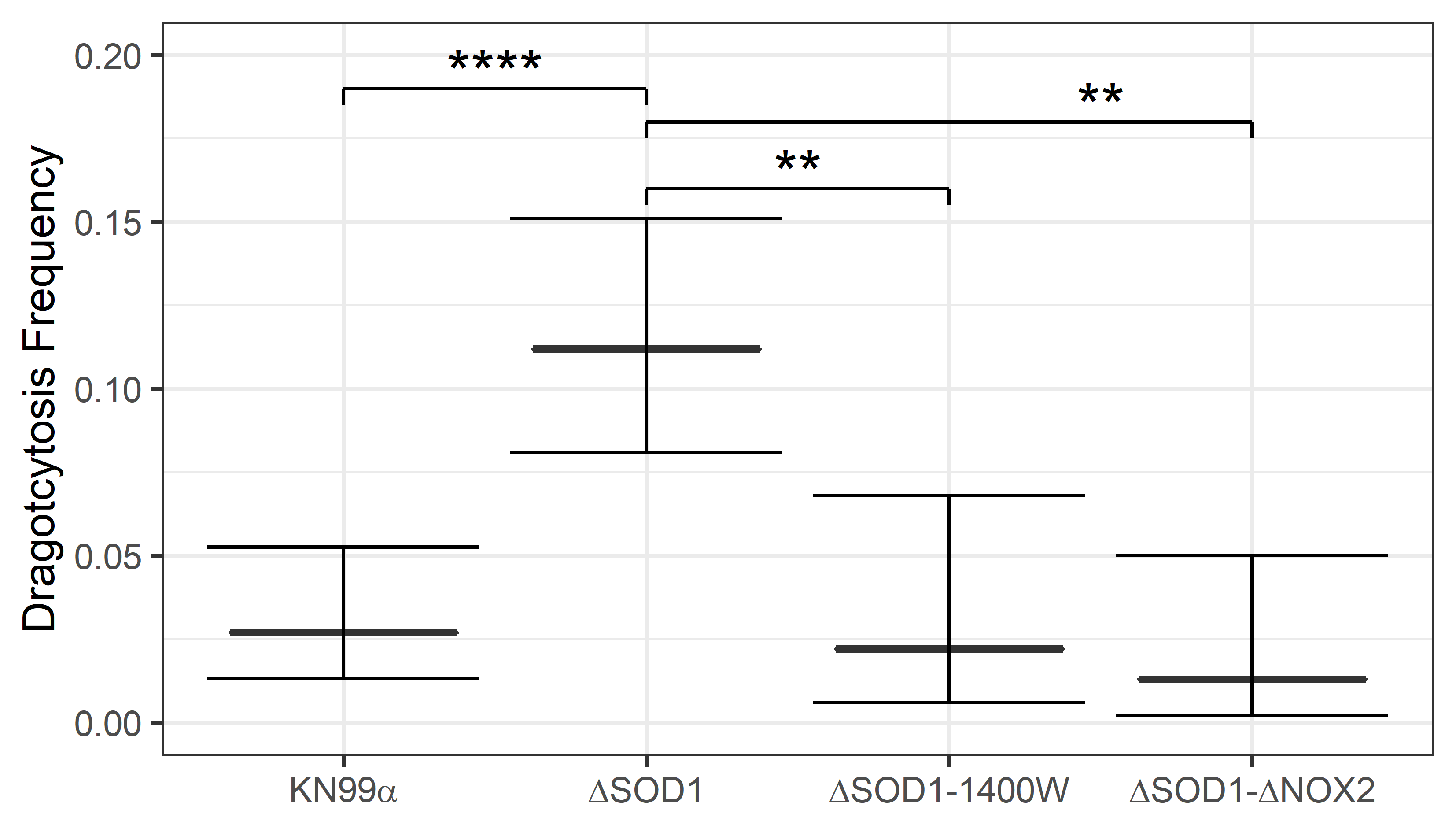

Supplement: S8 Fig — Both 1400W treatment and ΔNOX2 macrophage infection eliminate the increased Dragotcytosis phenotype, returning Dragotcytosis frequency to wild-type level. ** and **** represent P < 0.01 and 0.0001 via test of equal proportions with Bonferroni multiple hypothesis correction. Boxplots signify median with 95% confidence interval tails. For each experiment, data was collected for at least three independent experiments and with n of 333, 340, 136, and 158 infected macrophages for KN99α, ΔSOD1, ΔSOD1-1400W, and ΔSOD1-ΔNOX2, respectively. (TIFF) [file ppat.1010697.s008.tiff]

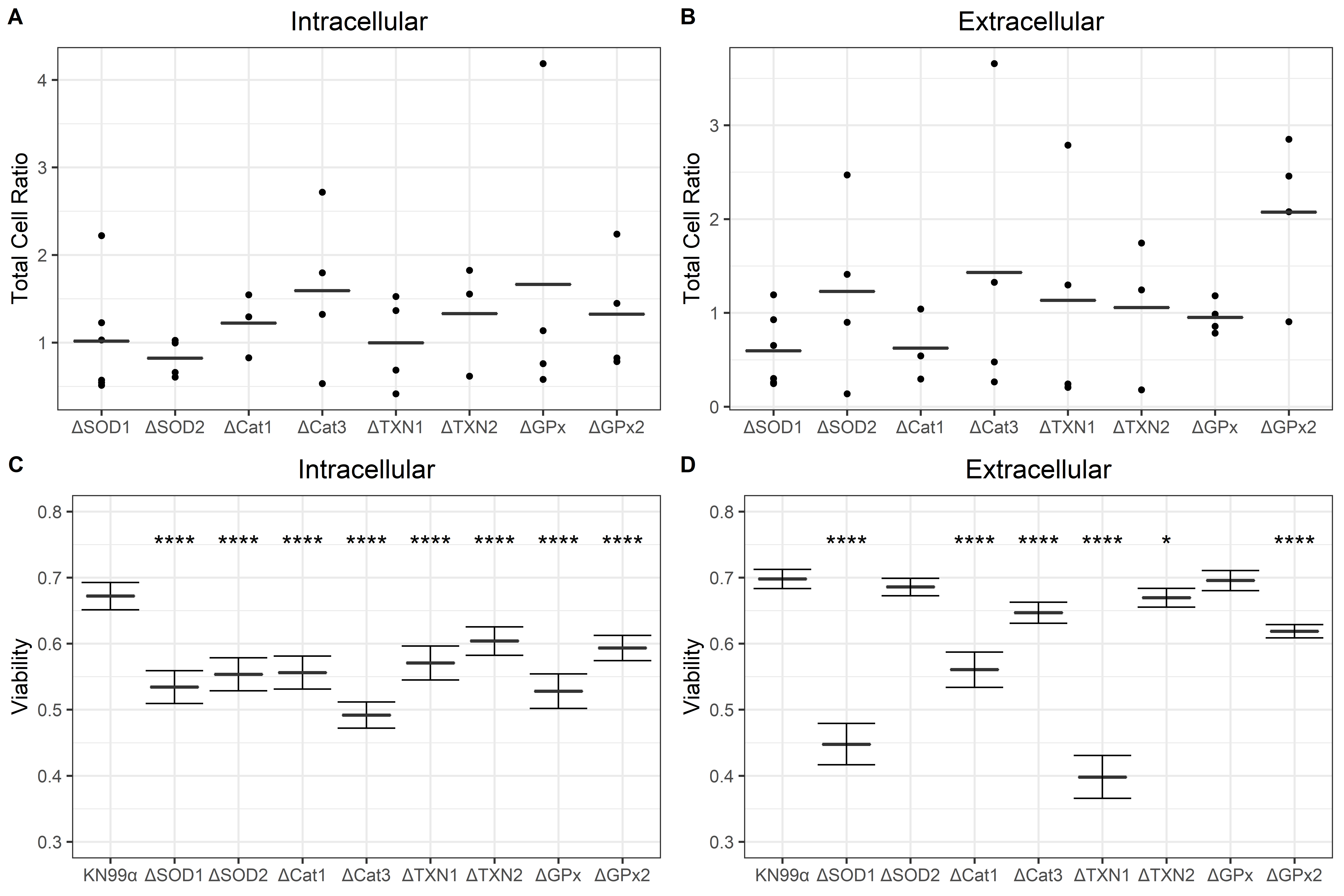

Supplement: S9 Fig — A. Total yeast cells recovered intracellularly normalized to the number of recovered wild-type cells. B. Total yeast cells recovered extracellularly normalized to the number of recovered wild-type cells. Total cell counts were measured via hemocytometer count and no significant deviation was detected by two-tailed t-tests with Bonferroni correction. Samples were measured from 3–4 independent experiments. C. Viability of yeast recovered intracellularly. D. Viability of yeast recovered extracellularly. Viability was measured via trypan blue stain and compared to wild type via test of equal proportions with Bonferroni correction. Samples were taken from three independent experiments with n of 2029, 1580, 1555, 1548, 2485, 1454, 2028, 1426, and 2583 yeast cells for KN99α, ΔSOD1, ΔSOD2, ΔCat1, ΔCat3, ΔTXN, ΔTXN2, ΔGPx, and ΔGPx2 respectively. * and **** denote P < 0.05 and 0.0001, respectively. (TIFF) [file ppat.1010697.s009.tiff]

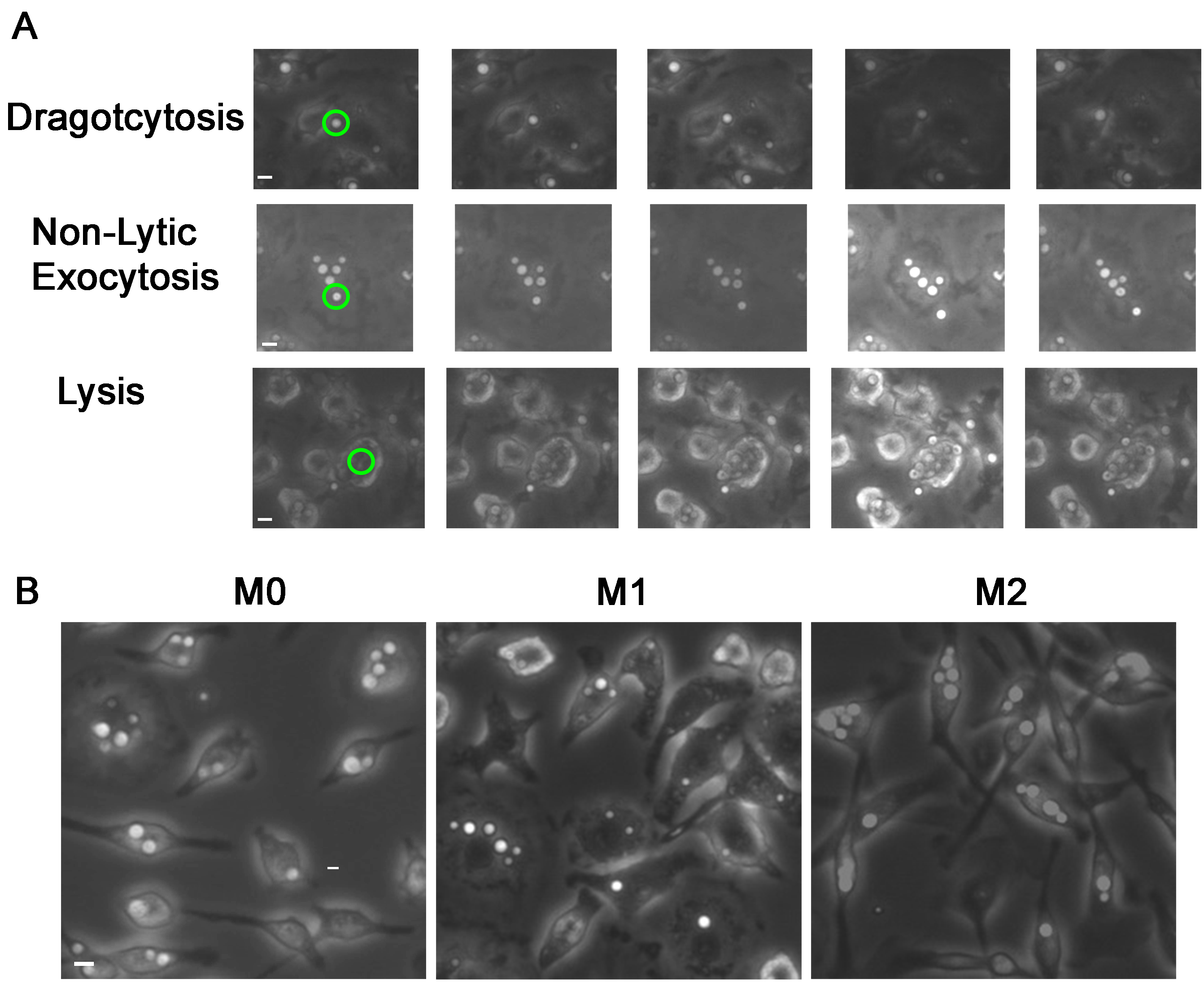

Supplement: S10 Fig — A. Examples of each type of host escape event taken from time lapse microscopy. The yeast undergoing each event is outlined in the first frame in green and each frame is 2 min apart. B. Representative images of each polarization type infected with C. neoformans. Scale bar represents 20 μm and is consistent across all images. (TIF) [file ppat.1010697.s010.tif]
