# Supplementary material for: Bet-hedging antimicrobial strategies in macrophage phagosome acidification drive the dynamics of Cryptococcus neoformans intracellular escape mechanisms
Source: PLoS Pathog. 2022 Jul 11;18(7):e1010697. doi: 10.1371/journal.ppat.1010697 (PMC9302974; doi:10.1371/journal.ppat.1010697)
Supplement: Attachment — Submitted filename: 2022-5-11_ReviewerResponses.docx [file ppat.1010697.s011.docx]

Dear Editor,

We are pleased that our manuscript received overall favorable reviews. The comments and criticisms from the reviewers were very helpful and led to additional experiments that have strengthened the paper and conclusions. We hope that the revised paper will be acceptable for publication. Below is a point-by-point response to reviewer comments.

Reviewer 1

Major

line 63-65. The authors claim that Dragotcytosis is favorable to Cn ... but if I understood the evidence correctly, it is in fact mutually beneficial, because it saves the host from death/lysis. It is an egress strategy similar in its consequences to "ejection" of Mycobacterium marinum from its amoeba host, Dictyostelium (PMID: 19325115 and PMID: 25646440)

Response:

We agree with the reviewer that there is more complexity and nuance to this hypothesis and have updated our manuscript to include this information.

Action Taken:

Manuscript text has been updated and references included.

line 271-272. It is very counterintuitive that the frequency of dissemination by Dragotcytosis is claimed to be independent of cell density. Every cell-to-cell dissemination process has to be proportional to cell density. Please, explain.

Response:

The total number of Dragotcytosis events are indeed proportional to cell density, what we observed was that the frequency of Dragotcytosis events did not correlate with cell density. We just wanted to ensure there was not an additional effect of population density on Dragotcytosis frequency aside from the expected increase in total number of events due to increased number of proximal macrophages. We have made our language more precise to reflect this.

Action Taken:

Manuscript text has been updated.

line 297 and below. I am uncomfortable with the claims and experimental strategies. The authors mention correctly that the fate and biochemical features of individual phagosomes are intrinsically phenotypically heterogeneous, but their study only quantifies outcomes of Cn infection in populations of M1/M2/M0 macrophages. Where is the cell-to-cell phenotypic heterogeneity? Some rephrasing and attention to this would be welcome.

Response:

We had some difficulty in sorting out what was meant in this criticism. We believe the reviewer is referencing that our polarized macrophages are not exclusively M0, M1, or M2 but rather mixes of each? Assuming we understand correctly, we agree and are careful to write ‘M# skewed’ to make it clear we are talking about skewed but mixed populations. Ensuring a population of 100% M2 or M1 macrophages, is technically difficult and even with the best methodologies one is likely to have heterogeneity since these phenotypes occur as a cellular distribution. We believe that that mixed populations more closely resemble an *in vivo* infection in which both populations would be present but in different proportions according to genetic background and other factors. We have reviewed the manuscript and made sure to update our language and ensure this is as clear as possible.

Action taken:

Text in the manuscript has been edited to make this distinction clearer, and to emphasize that we are talking about skewed populations.

line 320 and below. Similarly, it is claimed that Cn infection "skews towards M2 polarisation", but there are many plausible mechanisms for this bias. One could be "pushing" macrophages to differentiate into M2 state, with an imbalance between M1 and M2. Alternatively, Cn infection might lead to equal numbers of M1 and M2, but for example lead to rapid elimination of infected M1 cells. Please, clarify.

Response:

We agree that we are not proposing a mechanism for how the shift to M2 is achieved. We have updated the language of the manuscript to more clearly emphasize that we see a correlation but that we aren’t sure how the live *C. neoformans* achieve this skew.

Action taken:

The manuscript text has been updated to clarify this point and emphasize that we are not trying to suggest *C. neoformans* are solely actively responsible for shifting this skew.

line 329 and below. The authors claim that the chemical features of the host phagosome (pH) is driving/impacting Dragotcytosis, and to demonstrate this first use drugs/chemicals that perturb them. I really fear that the conditions used also have an impact directly on Cn, not only the host. And this is confirmed by the use of Amphotericin that targets only and directly CN ... and leads to a similar impact on Dragotcytosis. Please, disentangle and clarify.

Response:

Our assumption was that the amphotericin is either too strong (inhibits crypto before they have a chance to Dragotcytose) or damages them in a way independent of Dragotcytosis triggering mechanisms. Both drugs mechanism of action involve inhibition of ergosterol synthesis, distinct from oxidative damage and pH stress. We also wanted to emphasize that care must be taken to ensure experiments are carefully curated to detect lowered Dragotcytosis frequency due to relevant pathways rather than just dead *C. neoformans*. We have performed the requested growth curve experiments as suggested, adding a supplemental figure and updating the text to more clearly emphasize this point.

Action taken:

We have performed the requested growth curve experiment and included the results as a supplemental figure. The manuscript text has also been updated to ensure clarity.

The authors mention in Figure legends the statistical tests applied ... but there is no mention of the number of independent biological replicates and technical replicates. In addition, good practice would be to represent the data points, not only the boxes and violin envelopes.

Response: We agree that samples size and replicate information should be included and have updated the manuscript accordingly as well as converted boxplots to individual data points in experiments with low samples sizes, but respectfully disagree on the use of individual points in experiments with larger sample sizes. We believe visualizing all the data points results in 1. Overlapping points in high sample sizes which disrupts the ability to see individual points or 2. Pseudo random ‘jittering’ for visualization purposes which results in a misleading distribution, whereas box and violin plots include all relevant information on the distribution and statistics of a population. For graphs in which there is only an average line with bars we cannot graph individual points as they are z-tests of proportions in which each datum is a binary 1 or 0.

Action taken:

Figure legends and methods sections have been updated where necessary to include sample size and graphs have been updated to show individual points rather than boxplots where the sample size is low enough to not be misleading.

Minor

line 47, "lytic exocytosis" is non-sense. The authors likely mean "lytic egress".

Response:

The reviewer is correct on both counts.

Action taken:

We have updated the manuscript.

I am not completely convinced that the simulations (described from line 376 and below) really bring anything in terms of mechanistic validation. I cannot assess the rationality of the assumptions, and therefore, the soundness of the outcome. Figure 3 and the conclusions drawn are a bit esoteric to me.

Response: Simulations provide a way of testing the contribution of parameters. When simulations match experimental findings there is increased confidence that the relevant parameters have been identified and modeled appropriately. In our paper we designed a simulation to mathematically test a hypothesis that could explain the experimental data. While we agree that the correspondence between the simulation and observed results does not prove that the parameters considered are correct, the fact that the simulation produced results consistent with the experimental data gives us confidence that we are in the right track in thinking about this system.

Action taken:

The manuscript has been edited to increase clarity.

Similarly, I am not sure I can evaluate the validity of Figure 4C. An expert in simulations should maybe participate in this reviewing.

Response: We have edited the manuscript to increase clarity on these topics.

Action taken:

The manuscript has been edited to increase clarity.

Reviewer 2

Major

qPCR:

The authors should list and quote the primers used for SYBR green assay.

As a good practice, it is recommended to use at least two (but preferably 3) reference genes to determine more accurately the expression of specific genes (Vandesompele J, Preter K de, Pattyn F, Poppe B, Roy N van, Paepe A de, Speleman F. 2002. Accurate normalization of real-time quantitative RT-PCR data by geometric averaging of multiple internal control genes. Genome biology 3:RESEARCH0034. - Alanio A, Vernel-Pauillac F, Sturny-Leclère A, Dromer F. 2015. Cryptococcus neoformans Host Adaptation: Toward Biological Evidence of Dormancy. mBio 6:e02580-14.). Please add a reference on the primer used and perform an in silico validation of the primers in Primer BLAST (specificity of the primers for the tested genes).

Response:

We appreciate the reviewer’s suggestions and agree this data should be included. Thus we have updated the manuscript to include the requested information. All primers were targeted to the host macrophages and were commercially pre-optimized from Qiagen.

Action taken:

The manuscript has been updated to include qPCR primer information.

As capsule acts to buffer the acidification of the phagolysosome, how does this impact the final pH observed in the cells. Comparing the distribution of pH in beads-infected macrophage or capsulated infected or CAP mutant infected macrophage should be a nice addition to the paper. A nice experiment would be to use acapsular strain or hypocapsulated strain to potentially even boost the decrease of the pH and better uncover the increased of the dragotcytosis.

Response:

We have previously explored the relationship between bead infected and acapsular mutants in our most recent publication referenced in this paper. Indeed, both inert beads and acapsular *C. neoformans* mutants end up in a much higher proportion of lower pH phagolysosomes. Similarly, the effect of inducing capsule growth was previously studied by our lab, showing an expected overall increase in buffering potential. We have updated the text to make this clearer in the manuscript as we agree with the reviewer it is an important comparison to show.

Action taken:

We replicated capsule induced phagolysosomal pH measurements and added the results to a supplemental figure as well as updated the text to clearly reference these experiments and their previous results.

Fig 1C, in parallel to viability, it would be interesting to have the number of crypto cells counted or detected in each condition to see if macrophage polarization impacts proliferation as well.

Response:

We agree this would be a valuable comparison and have replicated our survival experiments to include total cell counts of both intracellular and extracellular *C. neoformans* at each time point.

Action taken:

We have performed the requested experiment and updated the text to include the results.

Line 309-310, it could be mentioned that dormant cells could be actin positive since expression of different genes have already been determined with actin as a reference gene (so the reference gene actin was expressed). It could be nice to see if part of the cryoto cells are dormant cells or if dormant cells put the system toward an increase dragotcytosis (or not)

Response:

We agree it is valuable to specifically investigate cell death. Thus, while replicating our survival experiments, we included a propidium iodide staining to specifically investigate cell death. As to dormancy we feel that this would be a separate study beyond the scope of this paper.

Action taken:

We have performed viability experiments with a cellular viability stain and included the results in the manuscript alongside an additional supplemental figure.

Minor

Is there a difference in the rate of dragotcytosis between H99 and KN99alpha? It should be interesting to get a figure on the potential difference between these two strains?

Response:

There is a large difference between H99 and KN99α rates of Dragotcytosis. The knockout library is generated from the KN99α strain, and we see rates of ~0.088 and ~0.027, respectively, with *P* = 0.00032 via test of equal proportions. The data is included in Figure 2 however the strains are separated by panel to make visualization of the other main comparisons easier, and we originally had KN99α labelled as ‘parental’.

Action taken:

We have updated the text to reference this comparison and explore the relationship between strains and updated the figure to increase clarify.

Reviewer 3

Major

The authors must provide images of cells undergoing Dragocytosis and of BMM (M0, M1, M2) infected with C. neoformans.

Response:

We can appreciate the reviewer’s desire for representative images and have taken individual frames from the time lapse microscopy experiments to provide the requested examples in a supplemental image.

Action taken:

We have prepared and included the requested images as a supplemental figure.

Figure 1: Viability is distinct from replication. The way the data is presented in Fig 1D, it is unclear whether and to which extent C. neoformans replicates in M0, M1, and M2 BMM. The authors should therefore include data on the replication of C. neoformans in M0, M1 and M2 BMM.

Response:

We agree with this reviewer and were able to address this while addressing a similar comment from reviewer 2. We replicated our survival experiments with the addition of total cell counts to investigate proliferation and propidium iodide staining to investigate cell death vs dormancy. The mutants did not replicate faster or to higher population levels but, as expected, they suffered reductions in viability compared to wild type cells.

Action taken:

The requested experiment has been performed and manuscript text updated to reflect this.

Figure 1, the authors hypothesize that NO buildup in the extracellular media is responsible for the inhibition of extracellular C. neoformans. It is simple to measure nitrites. Also, experiments can be performed with iNOS inhibitors.

Response:

The methods used in the paper to estimate NOS activity do function by measuring nitrites in the media as an estimation of NOS activity so Fig 1C does reflect nitrite levels. We agree with the suggestion to add iNOS inhibitors and have updated our results to include experiments using 1400W, a commercially available inhibitor, as well as the NOX-2 KO mouse experiments mentioned below. We also included 1400W in the growth curves requested by reviewer 1.

Action taken:

We have performed the requested experiment(s) and updated our manuscript accordingly.

Figure 2A: What is the impact of various treatments of macrophages (chloroquine, bafilomycin, minimal medium, 30C, etc) on the survival and replication of C. neoformans?

Response:

Our doses and treatments were chosen according to previous publications which noted inhibitory but not deadly concentrations/conditions, but we appreciate the robustness in showing growth curves supporting these assertions. In accordance with reviewer 1 we have performed additional growth curve experiments with each drug and have updated the manuscript for additional clarity.

Action taken:

We have performed the requested experiment and updated the manuscript accordingly.

Figure 2B: what is the survival/growth in macrophages of the C. neoformans mutants (SOD, Cat, TNX, GPx, etc) used to assess the impact of oxidative stress on Dragocytosis? Once released out of the macrophage, what are the survival rates of those mutants compared to WT C. neoformans?

Response:

We agree this would be a very interested experiment, especially to have alongside the wild-type H99 data of Figure 1. We replicated our survival experiments with each of the mutants compared to the respective parental KN99α strain and found, as expected, the mutants suffer reductions in viability across the board.

Action taken:

We performed the requested experiment and updated the manuscript accordingly.

The authors should take advantage of BMM from NOX2-ko mice to directly assess the impact of oxidative stress on Dragocytosis.

Response:

We agree with reviewer this is an interesting and valuable addition to the manuscript and have performed infection experiments on NOX2-KO mice from Jackson Laboratories. As expected, Dragotcytosis was reduced in both wild type H99 as well as the ΔSOD1 strain.

Action taken:

The requested experiment was performed, and the manuscript updated.

What are the C. neoformans molecules/pathways responsible for the triggering of Dragotcytosis? What about including secretion mutants of C. neoformans in this study to determine whether secreted factors are required for Dragotcytosis.

Response:

We appreciate the reviewer’s curiosity on the molecular pathways as elucidating this is our overall goal and will ideally be the next manuscript in this project. The discoveries in this manuscript have provided us a foundation of the triggers of Dragotcytosis, which we have been working on expanding and using to identify key molecular players in the process. Ongoing work in our laboratory is screening mutant libraries for effects on Dragotcytosis and we have candidate genes that will be reported elsewhere. Inclusion of that work, with all the attendant characterization, controls, etc. would be overwhelming in one manuscript.

Action taken:

We have updated the discussion section with additional text to reference these ideas.
